# Hanuman Complex And its Resolution : An Illustration of Psychotherapy from Indian mythology

**Published:** 2004

**Authors:** N N Wig

**Affiliations:** * Emeritus Professor of Psychiatry, PGIMER, Chandigarh. Residence: 279, Sector 6, Panchkula - 134109. nnwig@glide.net.in

## Abstract

The rich heritage of Indian mythology has been very little explored and used in psychotherapy in India. The present article deals with the story of Hanuman. How he lost the knowledge about his power to fly due to a childhood curse by Rishis and how he regained his powers when reminded by Jambavan during a crucial mission in search of Queen Sita, is the subject of author′s description of Hanuman complex and its resolution. The author has often used this story in helping patients in psychotherapy as well as in teaching medical doctors and trainees in psychiatry. A plea is made for wider use of stories from Indian mythology in psychiatric practice.


*Indian Journal of Psychiatry, 2004, 46(I)25-28*

### Hanuman Complex and its Resolution: An illustration of psychotherapy from Indian mythology

**N.N. Wig***

**Abstract**

The rich heritage of Indian mythology has been very little explored and used in psychotherapy in India. The present article deals with the story of Hanuman. How he lost the knowledge about his power to fly due to a childhood curse by Rishis and how he regained his powers when reminded by Jambavan during a crucial mission in search of Queen Sita, is the subject of author's description of Hanuman complex and its resolution. The author has often used this story in helping patients in psychotherapy as well as in teaching medical doctors and trainees in psychiatry. A plea is made for wider use of stories from Indian mythology in psychiatric practice.

### INTRODUCTION

Like most other children in India, Hanuman was my favourite hero in childhood. We would listen with wonder the story of his heroic deeds in the service of Lord Rama from the elders in the family. It was fascinating to hear how he could fly like a bird in the air carrying mountains. Knowing, that he was essentially a monkey made him particularly adorable to us as children. His pranks always had a child-like quality.

When I grew a little older I started reading in more detail his story from the Ramayana and other sources. I learnt that although Hanuman was always considered wise and brave, he did not fully realize his potential to fly and do other great deeds till much later when he was on a mission to search for queen Sita who was kidnapped by Ravana and taken to Lanka. This part of the story, how Hanuman did not know his full powers till reminded by Jambavan, became the nucleus of my theme of what I have called "Hanuman Complex". I have used this idea many times as a psychiatrist in treating patients. I have also used this story in teaching doctors and also in many public lectures. Though I have talked about "Hanuman Complex" for over forty years, somehow I never came to writing it formally for an academic publication. The present article is an attempt to fill that lacuna.

The original story appears in Valmiki's Ramayana. Tulsidasa's Ramayana (Ram Charit Manas) has only a much shorter version of the same story without reference to Hanuman's divine origin. Tamil Kamba Ramayana contains the same Valmiki's version but in much less detail. The readers in English can see the original story in the Ramayana by C. Rajagopalachari published by Bharatiya Vidya Bhawan. (Rajagopalachari 1983) A fuller version is available in the original Valmiki Ramayana translated by Makhan Lal Sen and first published in Calcutta in 1927. (Sen 1989) Since Hanuman is a very popular mythological figure, there are also other versions of the story with minor variations in other Puranas & folk literature. In Hindi, a very rich source of Hanuman legends is KALYAN magazine's "SRI HANUMNAN-ANK" first published by Gita Press, Gorakhpur in 1975. It contains extensive references to all the stories about Hanuman as given in various Puranas as well as in Ramayana by different authors. The essential story of Hanuman which I have picked up from various sources and on which I have based by psychological concept of "Hanuman Complex" is narrated below.

### The Story of Hanuman

Hanuman was known as the son of monkey King Kesari and mother Anjana but in fact he had a divine birth. The mother princess Anjana was one day roaming in hills when God of wind (Pawan or Vayu or Marut) spotted her and was captivated by her beauty. As a result of this union, Hanuman was born who like his father, God of wind, had the powers to fly and reach any part of the earth. As an infant he once flew up to catch the sun, when the king of heavens Indra, got annoyed and threw his thunderbolt at him. Hanuman survived the thunderbolt but it broke his jaw – that is why the name "Hanuman". (In Sanskrit HANUMAN or HANUMAT means prominent jaw). The other name for Hanuman, popular in South India is ANJANEYA or son of Anjana.

The God of wind (Vayu) got very upset with this action of Indra to his son Hanuman and stopped the wind from blowing. The life came to a standstill on earth. Indra apologized and along with other gods bestowed many boons on child Hanuman.

***Emeritus Professor of Psychiatry, PGIMER, Chandigarh. Residence: 279, Sector 6, Panchkula – 134109. Email : nnwig@glide.net.in**

Now with these new powers, Hanuman became very mischievous as a child. He would pick up articles of Rishis (holy men) while they are in prayers and fly away. This would greatly annoy the Rishis and they threw a curse at Hanuman that he will lose his power to fly. Hanuman was naturally very upset and told mother Anjana about it who pleaded with the Rishis for forgiveness. Finally, the Rishis relented and modified the curse to say that Hanuman will not lose his divine power to fly but he will henceforth lose the knowledge about his powers till reminded about it at an appropriate time by some wise man.

The story then moves to Ramayana where king Sugreeva, a monkey king and friend of Lord Rama, has sent a mission to search for Queen Sita, wife of Lord Rama, who had been kidnapped by demon Ravana to Lanka. The monkey party headed by crown prince Angad, Hanuman and others reach Land's-end and face the sea across which is the island of Lanka. They are all quite despaired to see the intervening sea. There is hurried consultation on what to do. Some members of the mission say they may leap some distance but not all the way to Lanka. Angad says he may perhaps succeed in reaching Lanka but may not have enough power to come back. Jambavan, an aged bear and the senior-most member of the party says he could have done it in his youth but not now in his old age. Then Jambavan turns to Hanuman and says, "Why are you sitting silent and dejected in a corner? Do you know who you really are? You are "Pawan Putra" – son of God of wind. You have the power to fly and reach any corner of the earth. Unfortunately, you are not aware of your own powers". Jambavan then narrates to Hanuman the story of his birth and childhood curse. Listening to this Hanuman gets back his powers and confidence. He assumes his great size and flies to Lanka, meets Sita and the story of Ramayana continues. In the further story, Hanuman does many more heroic deeds like bringing "Sanjivini" herbs from the Himalayas for the revival of Lakshmana and so on, but it is the intervention by Jambavan which transforms Hanuman into a great hero for the rest of the Ramayana.

### The Psychological Implications

As I have said in the beginning of this article, I have called this story of Hanuman not knowing his true potential, as "Hanuman Complex". I use this mythological tale to make two points:

1. To a patient who has lost confidence and who feels unable to meet life's challenges, I relate this story. Most of the patients have already known it. I point out that the power to change his life rests within him. He has temporarily lost the knowledge of his own powers due to his illness, due to this veil of ignorance. Like Hanuman he has to shake off this diffidence and realize his true potential. The golden Lanka lies across the sea and he has the power to reach there.
2. To the doctors in training I narrate this story to emphasize that "when you do psychotherapy, do not assume that power to change the life of the patient lies with you. In fact, the potential to change rests with the patient who has temporarily like Hanuman, lost it. It is your job as a therapist (like Jambavan) to restore this power back to the patient."

### Use of Indian Mythology in Psychotherapy

The Indian mythology is one of the richest mythologies in the world. Furthermore, unlike Greek, Roman, Egyptian or other great mythologies of the past, which are extinct now, the Indian mythological tradition is very much active and is part of the daily life of Indian people. The newer religions in Greece, Rome, Egypt or Mesopotamia have replaced the ancient religions based on mythology and once powerful mythological figures like Zeus or Aphrodite or Osiris and Isis are today to be found only on books and museums. In India, on the other hand, there has been a continuity of religious tradition for the last five thousand years. The ancient mythological figures like Shiva or Vishnu, Rama or Krishna, Ganesha or Durga are worshipped everyday all over the country. For an Indian, Ramayana or Mahabharata are not merely books of old epic stories like Homer's Iliad or Odyssey in Greece, but are models for day-to-day life and behaviour. Hence mythological stories have tremendous power and hold over Indian people. Religious teachers regularly use these stories to exhort listeners to modify their behaviour. It is surprising and sad that we, the mental health professionals make so little use of them. In Psychiatry we have incorporated the psychoanalytic concepts of Oedipus complex or Electra complex for psychotherapy, which hardly make an impact on our people, while our own rich heritage of mythology remains untapped or unused.

For the last few decades there is growing realization that for better practice of psychiatry we must make more use of the rich Indian philosophical and religious traditions. Prof. N.C. Surya of Bangalore was one of the early thinkers to draw attention to this (Wig 1996). Prof. N.S. Vahia of Bombay was another pioneer who through many articles popularized the use of Yoga for treatment of neurotic and psychosomatic disorders in India (Vahia 1973). Prof. A. Venkoba Rao has more than once beautifully written about the value of Srimad Bhagwad Gita in psychotherapy and for understanding the functions of mind (Venkoba Rao 1980, 2003). Unfortunately, the use of stories from Indian mythology have received relatively little attention. Erna

Hoch, a Swiss psychiatrist working in India for many years, did suggest their use (Hoch 1977). Perhaps the largest study on Indian mythology has been done by Dr. Shamasundar of NIMHANS Bangalore (Shamasundar 1993). He collected a number of excerpts from various well-known sources like Ramayana, Mahabharata and Buddha Jataka tales etc. He submitted these excerpts to various professional and laypersons asking their opinion about their applicability in therapy. He finally chose nine examples to illustrate the usefulness of these extracts in his paper in Am J. Psychotherapy 1993. His focus seems to be on selecting stories with clear messages or "morals" for use in therapy.

Myths are not ordinary stories with morals at the end. The power of the myth as Joseph Campbell has so well argued in many of his books lies not so much in its obvious content but in its subconscious message, something similar to what happens in a vivid dream (J.Campbell 1988). Carl Jung has stressed the same point in his book Man and His symbols (Jung 1990)

In recent years, in Europe especially in Scandinavian countries like Norway, Finland etc. many therapists are increasingly using stories, metaphors and allegories from their respective cultures. A new term "Narrative Psychotherapy" has been coined to describe these developments (Pakaslahti 2003). In USA, the work of Milton Erickson is well known for use of narrative and metaphor in patient work (Pakaslahti 2003). Nossrat Peseschkian from Germany who uses Persian Sufi tradition in his work has suggested the term "Positive Psychotherapy" to describe this. In a paper at World Congress of Social Psychiatry at Agra in 2001, he advocated the application of stories, fables, wisdoms and myths in such psychotherapy (Paseschkian 2001).

The story of Hanuman and his heroic deeds in the service of Lord Rama is known throughout India, may it be among the villagers in Rajasthan or tribals in Orissa. His temples are present in almost every village or town in India. The day Tuesday is particularly associated with his name when the devotees (especially students near the examinations) throng his temples. Hanuman Chalisa, a brief poem of forty couplets attributed to great poet Tulsidasa, describing Hanuman's super powers and achievements, is widely recited in all Hindu homes among the Hindi knowing people. Hanuman is particularly worshipped for quick relief from sufferings of all kind, that is why he is also called "Sankat Mochan" – or remover of suffering.

Hanuman's Balaji temple in Rajasthan is famous for the treatment of mentally ill of all kind. A number of mental health professionals from India and abroad have written about this famous temple (Satija et al 1981, Kakkar 1982, Pakaslahti 1998).

An important aspect of the concept of 'Hanuman Complex' is that it is not conceived only in negative terms, as are most other psychological "complexes" in Western psychotherapy. In the Indian story of Hanuman, there is not only reference to a psychological problem but also there is a way out to a solution in terms of psychotherapy by Jambavan.

The fame of Hanuman is not only confined to the shores of India. I quote from a recent letter by Dr. Pakaslahti about his views on the psychological implications of the story of Hanuman. Dr. Antti Pakaslahti is a psychotherapist & Docent (Associate Professor) of Psychiatry at the University of Oulu in Finland.

"As a psychotherapist, I see in Hanuman's story an allegory, a series of metaphorical images of a young one's psychological and social growth. The plucky little monkey boy who reaches for the sun – may be not just due to mere infantile spatial miscalculations but more probably on some symbolical level – may be claiming his rights for the brightness of childhood, the intrinsic right of every little one. May be there was a danger from which adults had to protect him, in this case Indra in a rather over-reacting way. Or may be, the haughty Indra was just angry at the menace of a lower creature's incursion into his realm? Anyway, the very breath of life stops when the courageous boy is too severely chastised. To be saved adults bestow on the boy their best gifts, marvelous boons and powers. Life breathes again and fresh winds can blow. Later comes adolescence and various silly mischiefs and the gifts are wasted by an unripe mind at the threshold of adulthood. The young man must be put to law and order by the rishis. It is better that he temporarily forgets about his superlative capabilities and learn more about more civilized ways. Finally, at the proper moment waking up into adulthood through wise advice by a mentor, finding one's true powers in order to assume adult tasks and duties. Jumping across the sea into unknown hostile territory to find, solace and save Sita, the queen in dire distress, kidnapped by a fierce demon, the enemy of culture and the good way of life. Despite his great male prowess, Hanuman is celibate, Sita is to him a mother not a beautiful lady. May be this is an image of sublimating animal force (or libido) into the service of culture and higher social values, including the well-being of gentle womenhood……" (Pakaslahti 2003)

I am sure mental health professionals in India will have many other thoughts about this beautiful story from Indian mythology, apart from the way I have looked at it in describing Hanuman Complex. In this article I am only making a plea for greater use of our rich heritage of mythology for our day to day work in psychiatry. People respond strongly to stories all over the world. Perhaps this is much more so in India.

### ACKNOWLEDGEMENT

I am grateful to Dr. Antti Pakaslahti of Finland for stimulating me to write this paper and for reading the manuscript and giving some very valuable suggestions.

### REFERENCES

Campbell Joseph with Bill Moyers (1988) The Power of Myth, Doubleday New York.

Hoch, E. (1977) Psychotherapy for the illiterate. In Arieti, s. (Ed) New Dimensions in Psychiatry – worldview, New York; John Wiley and Sons.

Jung, Carl G. (1950) Man and his symbols. Arkana – Penguin, London.

Kalyan Sri Hanuman Ank (Hindi) (1975), Kalyan Karyalay, Gita Press, Gorakhpur.

Kakkar S. (1986) Shamans, Mystics & Doctors. Oxford University Press. New Delhi.

Pakaslahti A. (1998). Family centered treatment of mental health problems at Balaji temple in Rajasthan. In: Parpola A. & Tenhunen s. (eds) Changing Patterns of Family and Kinship in South Asia. Finnish Oriental Society, Helsinki.

Pakaslahti Antti (2003) Personal communication.

Pesseschkian Nossrat (2001) The application of Wisdoms and Stories in positive psychotherapy. Paper presented at XVII World Congress of World Association for Social Psychiatry, Agra. Oct 27 – 31, 2001 (Abstract L-6).

Rajagopalachari, C. (1983) Ramayana published by Bharatiya Vidya Bhawan, Bombay. (1st Edition in 1951, 22nd Edition in 1983).

Satija DC, Singh D, Nathawat S.S, and Sharma V. (1981). A Psychiatric study of patients attending Mehandipur Balaji temple. Ind J Psychiatry 23 (3): 247 – 250.

Sen, Makhanlal (1989) Ramayana, Translated from the Original Valmiki. Present edition published by Rupa & Co. Calcutta 1989.

Shamasundar C. (1993) Therapeutic Wisdom in Indian Mythology. Am J Psychotherapy 47 (3) 443 – 449.

Vahia, N.S., Vinekar S.L., Doongaji D.R. (1966) Brit J Psychiatry 112, 1089 – 1092.

Venkoba Rao, A. (1980) Gita and Mental Sciences. Ind J Psychiatry, 22, 19 – 31.

Venkoba Rao, A. (2002) Mind in Indian Philosophy. Ind J Psychiatry, 44 (4), 315 – 325.

Wig, N.N. (1996) The Pioneers of Indian Psychiatry – Dr. N.C. Surya – Ind J Psychiatry 38 (1) 2 – 8.

